# OnkoRiskNET: a multicenter, interdisciplinary, telemedicine-based model to improve care for patients with a genetic tumor risk syndrome

**DOI:** 10.1186/s12913-022-08172-2

**Published:** 2022-06-21

**Authors:** Johanna Tecklenburg, Beate Vajen, Susanne Morlot, Petra Anders, Paula Memenga, Elena Link, Eva Baumann, Sarah Wölffling, Evelin Schröck, Anke Katharina Bergmann, Brigitte Schlegelberger

**Affiliations:** 1 Department of Human Genetics, Hannover Medical School, Hannover, Germany; 2 Department of Journalism and Communication Research, Drama and Media, Hanover University of Music, Hannover, Germany; 3 Department of Human Genetics, University Hospital Carl Gustav Carus Dresden, Dresden, Germany

**Keywords:** Genetic tumor risk syndrome, Genetic counseling, Telemedicine

## Abstract

**Background:**

Genetic tumor risk syndromes are responsible for at least five to ten percent of the 4 million cases of cancer diagnosed in Europe every year. Currently, the care of oncological patients suffers from a lack of specialists in medical genetics and also a lack of access to genetic care in rural areas and structured care pathways between oncologists and medical geneticists. As a result, genetic tumor risk syndromes are underdiagnosed with potentially fatal consequences for patients and their families.

**Methods:**

The OnkoRiskNET study is supported by a grant from the Federal Joint Committee of the Federal Republic of Germany. The study will include 2,000 oncological index patients from oncology practices in Lower Saxony and Saxony after the start of the study in July 2021. Randomization is carried out by means of a stepped wedge design at the level of the practices. Patients either go through routine care or the new form of care with structured cooperation between medical geneticists and oncologists, case management and the use of telemedical genetic counseling. Using a mixed-methods approach, the following parameters will be evaluated in the control and intervention group: (1) Conducted genetic counseling sessions by patients with suspected tumor risk syndrome and their first degree relatives; (2) Patient satisfaction and psychological distress after genetic counseling and testing; (3) Factors influencing the acceptance and experience of telemedical genetic counseling; (4) Satisfaction of oncologists and medical genetics with the structured pathway; (5) Cost efficiency of the new form of care.

**Discussion:**

OnkoRiskNET aims to close the gap in care through the formation of a cooperation network between practicing oncologists and specialists in medical genetics and the use of telemedical genetic counseling, thereby, increasing the diagnostic rate in genetic tumor risk syndromes and serving as a model for future genetic care in Germany.

**Trial registration:**

Trial was registered on 01.12.2021 in the German Clinical Trial Register (https://trialsearch.who.int/) with the DRKS-ID: DRKS00026679. Title: Cooperation network for the provision of local care for patients and families with a genetic tumour risk syndrome. Trial acronym: OnkoRiskNET. Protocol version 1.1.

**Supplementary Information:**

The online version contains supplementary material available at 10.1186/s12913-022-08172-2.

## Background

The discovery of inherited pathogenic variants that are responsible for an increased risk of cancer has opened a new field in cancer medicine. The diagnosis of a genetic tumor risk syndrome (TRS) enables personalized therapy, aftercare and prevention and the identification of further affected family members. Up to the present, more than 100 cancer predisposition genes and their association with tumor risk syndromes have been identified [1]. TRSs are responsible for at least five to ten percent of the 4 million cases of cancer diagnosed in Europe and of 500,000 cases of cancer diagnosed in Germany every year [2].

In those affected, characteristic tumors often occur at a young age and are significantly more frequent than in the general population. The risk of recurrent, independent tumors is also increased. Since the pathogenic variant can be passed on within the family, family members have an increased risk of developing cancer. A TRS can be diagnosed by analyzing the family history, calculating the risk using algorithms and by genetic testing. If pathogenic genetic variants are detected, specific therapies are available (e.g. PARP-inhibitors in a variety of cancers including ovarian, breast, prostate and pancreatic cancer) as well as risk-adapted surveillance and risk-reducing operations. Thus, the diagnosis of a genetic tumor risk syndrome enables effective primary, secondary and tertiary prevention.

Currently, the care of oncological patients in Germany suffers from a lack of specialists in human genetics and a lack of structured interdisciplinary care for patients with a suspected genetic tumor risk syndrome. In Germany, only about 350 medical geneticists are currently available for genetic counseling for a population of 83,2 million [3]. Furthermore, there is a lack of access to genetic care in rural areas: 88% of medical genetic outpatient clinics and practices are located in cities with more than 100,000 inhabitants and 50% in cities with more than 500,000 inhabitants [4]. In previous studies, teleconsultations and virtual consultations in medical genetics have proven their value as indicated by high levels of user satisfaction and have shown no inferiority to in-person consultation in terms of psychosocial outcome [5]. The multicenter, interdisciplinary, telemedicine-based OnkoRiskNET study therefore, aims to close this gap by implementing a new form of care with a structured care program, the use of telemedical genetic counseling and the formation of a cooperation network between oncologists and medical geneticists.

## Methods

### Sample

The study will include 2,000 oncological index patients from oncology practices in Lower Saxony with around 8 million inhabitants and Saxony with around 4 million inhabitants. The study started in July 2021 and will end in March 2025. Inclusion criteria are: adult patients of participating oncologists with suspected TRS who suffered from cancer within 36 months before inclusion in the new form of care. Exclusion criteria are: patients with familial breast and ovarian cancer, because a model of integrated care for this indication between the participating institutes and most health insurance companies in Germany has already been set up. Privately insured patients and patients under the age of eighteen also cannot participate in the study. A „noninferiority “ power analysis for the primary outcome was conducted based on the following assumptions:

- The mean participation in genetic counseling of cancer patients in international comparison ranges from 70 [6] to 80% [7]. Here, we assume 70% of cancer patients will participate.
- The rate of utilization of a telemedicine consultation and 'in person' consultation does not differ [8].
- For the statistical test of 'non-inferiority' we assumed 5% significance (alpha = probability of type 1 error) and 80% power (1-beta, 20% probability of type 2 error).
- The threshold for 'non-inferiority' is 5%.
- We assumed that 350 patients on average visit an oncology practice per quarter [9]. In at least five to ten percent of these patients, a genetic tumor risk syndrome is suspected. Therefore, we assumed that we can recruit about 50 to 100 cancer patients per year. Over a three-year period, 150 to 300 patients per practice can be recruited. We assume a conservative estimate of 100 patients per practice over the project period.

### Sample size calculation

A sample in a classic parallel individual-randomized controlled trial would therefore have to have 1040 subjects in each arm – a total of 2080 in the entire experiment. We continue to assume that 30% of the participants will drop out prematurely – so 2971 (2080/0.7) participants would have to be recruited. Randomization is carried out by means of a stepped wedge design at the level of the practices. Therefore, an intra-cluster correlation (ICC) of 0.05, and an average of 100 patients per practice creates a design effect of 5.95, bringing the total number of participants to be recruited to 17,680. Through the stepped wedge approach, however, the number of participants can be reduced by re-examining test persons in each step of the experiment. Assuming 4 steps (i.e. transitions from control to intervention group, new recruitments in each step and an additional survey for the baseline survey), a design effect of 0.62 is created. The total number of participants to be recruited is thus 1838 in the stepped wedge approach for a power of 80%. In order to achieve this total number of cases, 18 medical practices with about 102 subjects each are necessary.

### Study design

**Fig. 1:**
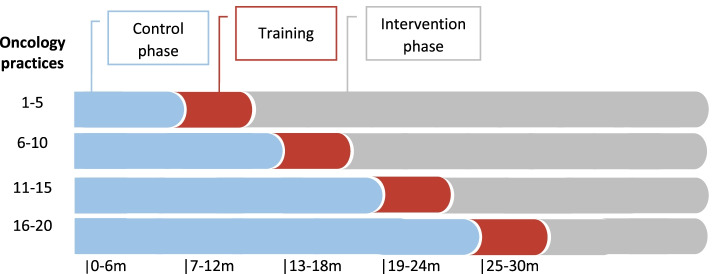
Stepped wedge design of the project OnkoRiskNET. Twenty oncology practices start with the control phase and will enter the intervention phase after training on TRS. The control phase reflects the standard care of patients with TRS in Germany. The intervention phase reflects the new form of care with closer cooperation between oncologists and medical geneticists and telemedical genetic counseling

Randomization is performed by an external independent research institute (INAV Institute, Berlin, Germany). All practices will continue the standard care under evaluation control phase, then with a time delay the transition to the intervention phase will take place (Fig. 1). The advantage of the stepped wedge design is that each practice benefits from the intervention. Before the intervention phase starts participating oncologists will receive training by medical geneticists. The trainings will focus on the basics of TRS, the collection and impact of an extended family history and possibilities and limitations of genetic testing. In addition, checklists or Standard Operating Procedures will be provided by the medical geneticists. In order to demonstrate the transferability of the new form of care, the study will not only be established in Lower Saxony but also in Saxony.

### Structured cooperation between medical geneticists and oncologists

**Fig. 2:**
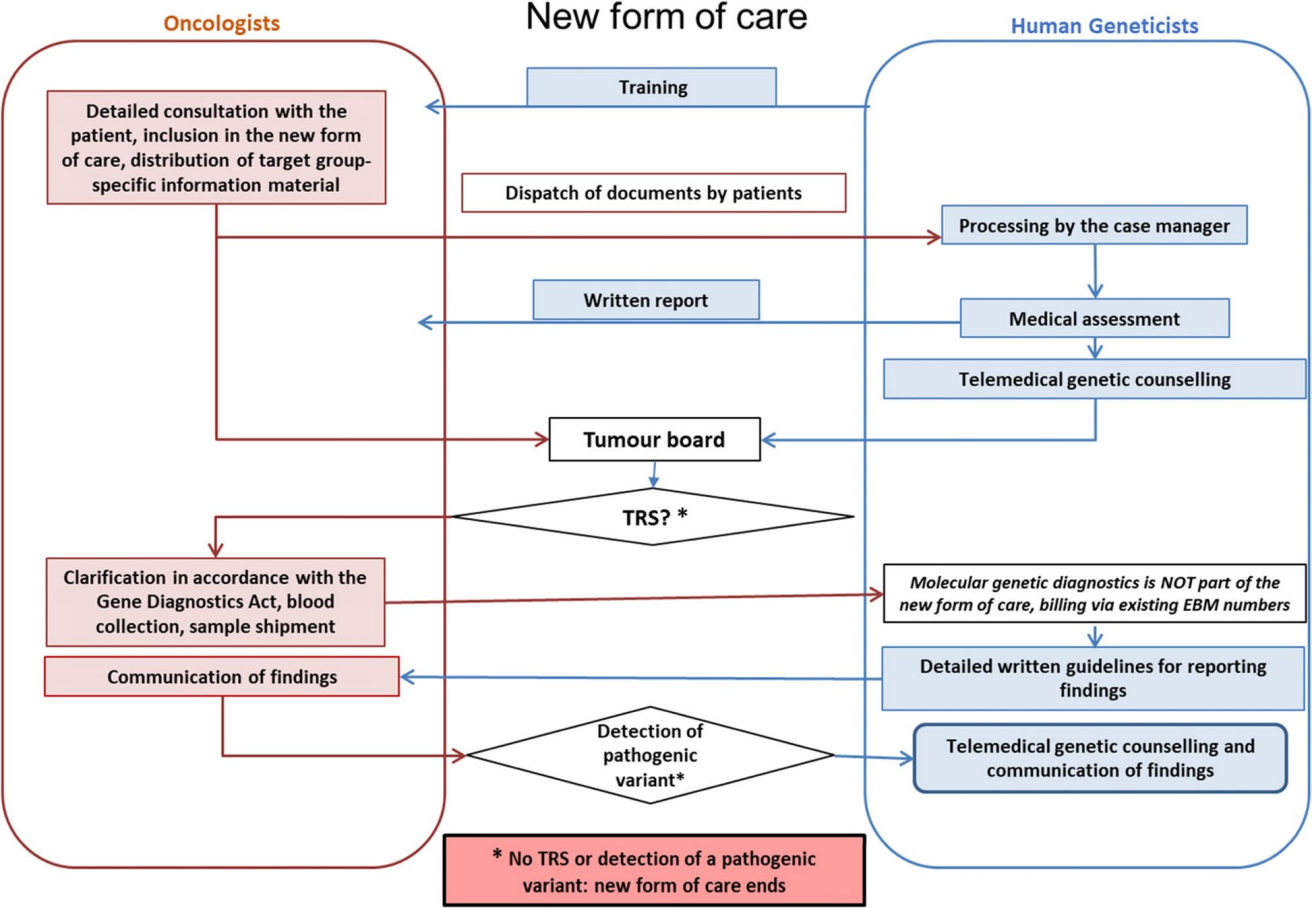
Characteristics of the new form of care. In contrast to the standard care of patients with TRS, the new form of care aims to implement telemedical genetic counseling and a closer cooperation between oncologists and medical geneticist of the Departments of Human Genetics

The new form of care is characterized by close contact and frequent exchange between oncologists and medical geneticists (Fig. 2). The patients will be included in the study by the participating oncologists. They will receive specific written information for their target group. Patient consent, family history and patient records will be sent to the participating medical geneticists. These are reviewed and evaluated by the case management and subsequently by medical geneticists. Patients then receive telemedical genetic counseling. The cases are discussed in weekly internal case discussions. Oncologists have the opportunity to participate via video telephony. Patients and oncologists receive a written evaluation of the findings. In case of suspected TRS, the genetic testing is prompted by the oncologist. Oncologists receive a detailed guide with interpretation of the results of genetic testing and its clinical consequences for the patients and family members. Based on this, the oncologists inform the patients about the findings in their practice. The new form of care ends if no TRS has been diagnosed or no pathogenic variant has been detected. In case of detection of a pathogenic variant, a second telemedical genetic counseling will be performed by medical geneticists.

### Case management

In the Department of Human Genetics, a coordination office for the project has been established. Case managers coordinate and supervise the scheduling of appointments and check the submitted documents for completeness. If necessary, further information is obtained from the patients. The information on existing cancer diagnoses in the family is summarized and a pedigree is created. This is intended to reduce the work on the part of medical geneticists, so that they have more resources for the actual genetic counseling.

The case managers also facilitate communication and ensure that no information is lost in the exchange between oncologists, medical geneticists and patients. Furthermore, they are in constant interaction with the oncologists in order to achieve adequate participant enrolment to reach target sample size. The case managers offer continuous support to patients over the entire participation period and monitor the adherence.

### Telemedical genetic counseling

In case of suspected TRS, a telemedical genetic counseling takes place. Here, patients are informed about genetic risk assessment, receive recommendations for genetic diagnostics as well as information on preventive measures. Telemedical genetic counseling is preferably carried out via video consultation directly with the patients. This is carried out by a provider of telemedical software, which is certified by the German National Association of Statutory Health Insurance Physicians (KBV Kassenärztliche Bundesvereinigung) and meets its data protection requirements. If telemedical genetic counseling is not possible due to technical or infrastructural reasons, there is the possibility of a telephone consultation. Patients and treating oncologists receive a written summary of the counseling. In case of detection of a pathogenic variant, a further detailed telemedical genetic consultation on the significance and consequences of the examination result is carried out by medical geneticists mainly focusing on psychosocial aspects. The further coordination of therapy, surveillance, preventive measures and care is carried out by the oncologists as part of the standard care.

### Evaluation

**Fig. 3:**
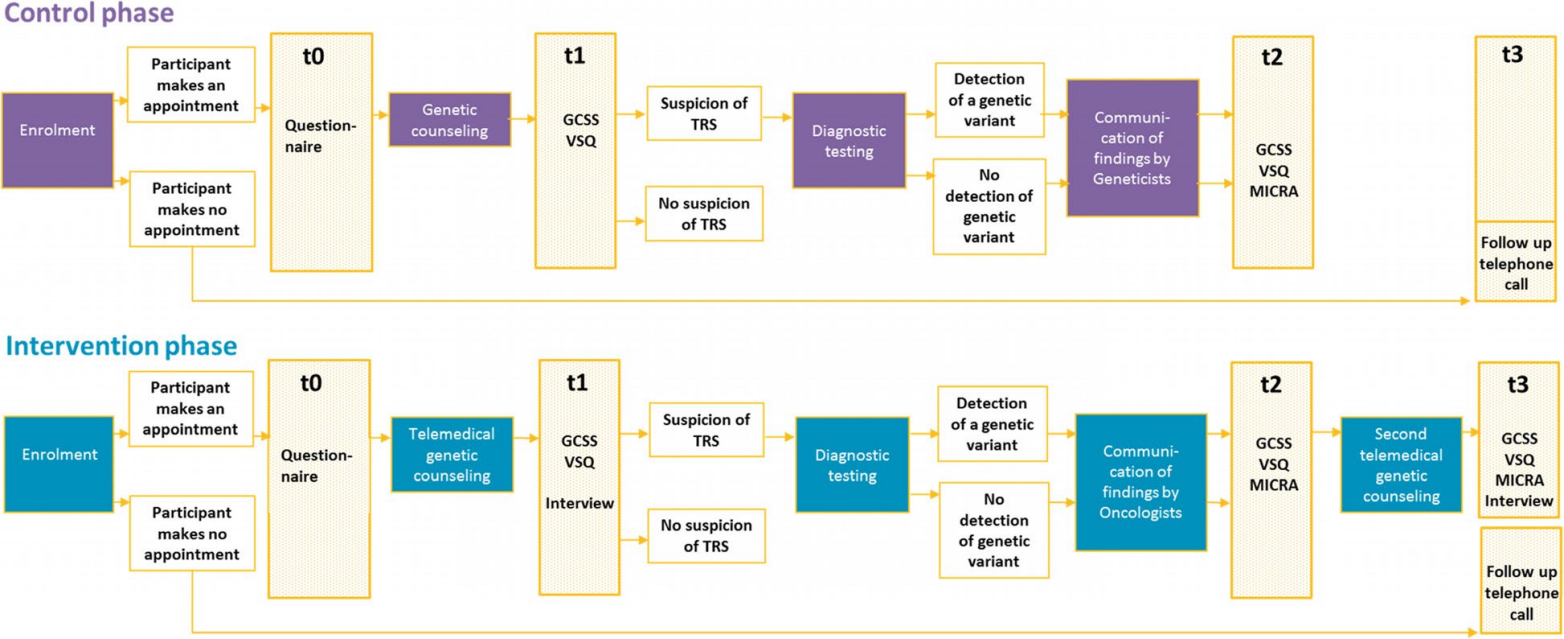
Schedule of enrolment, interventions and assessments. In the control phase participants make an appointment in the Departments of Human Genetics Hannover or Dresden after enrolment. They receive a questionnaire about socio demographic data and their expectations of the genetic counseling. One week after genetic counseling participants receive the GCSS and VSQ questionnaires. Participants with a suspicion of TRS get diagnostic testing. Test results are communicated by Geneticists. Afterwards participants are asked to fill out the questionnaires GCSS, VSQ and MICRA. If participants have been enrolled but no consultation has been recorded, the patient is asked about the non-utilization by a follow-up telephone call (t3). In the invention phase telemedical genetic counseling takes place. Oncologists communicate the test results of genetic testing and a second telemedical genetic counseling takes place

The endpoints will be evaluated by the external independent research institute (INAV Institute, Berlin, Germany). The schedule of enrolment, interventions and assessments is shown in Fig. 3. Using a mixed-methods approach, the following parameters are evaluated in the control and intervention group: (1) Conducted genetic counseling sessions by patients with suspected TRS and their first degree relatives; (2) Patient satisfaction and psychological distress after genetic counseling and testing; (3) Factors influencing the acceptance and experience of telemedical genetic counseling; (4) Satisfaction of oncologists and medical geneticists with the structured pathway; (5) Cost efficiency of the new form of care.

1. **Conducted genetic counseling sessions by patients with suspected TRS and their first-degree relatives**
  Acceptance of the offer of genetic counseling is determined by comparing the number of patients who received a referral from their oncologists to the Departments of Human Genetics with the number of patients who actually scheduled an appointment. The participating oncologists document their recommendation for referral and send patient data to the respective Department of Human Genetics in Hannover or Dresden. The Departments keep track whether a consultation has actually taken place.
  If no consultation has been recorded, the patient is asked about the non-utilization by a follow-up telephone call. The call is made shortly before the end of the study to avoid the call itself having an impact. In the intervention group, utilization is recorded as participation in telemedical genetic counseling.
  The utilization of genetic counseling by relatives at risk is recorded by the Departments of Human Genetics analogously.
2. **Patient satisfaction and psychological distress after genetic counseling and testing**
  Quantitative and qualitative methods are used to measure patient satisfaction. For quantitative analysis, two validated questionnaires are used, the Genetic Counseling Satisfaction Scale (GCSS) [10] and the Visit-Specific Satisfaction Questionnaire (VSQ) [11]. In addition, qualitative, semi-structured individual interviews with patients are planned after each telemedical genetic counseling session. The focus of the interviews is the acceptance and subjective experience of the technologically mediated form of counseling. Furthermore, patient information material is examined with regard to comprehensibility and usefulness. If a second telemedical genetic counseling on the meaning and consequences of positive test result is carried out, the satisfaction with the psychosocial counseling is the focus of the survey. Psychological distress after genetic diagnosis is assessed with a validated questionnaire, the Multidimensional Impact of Cancer Risk Assessment (MICRA) [12], which has specifically been developed for this context.
  The values of the intervention group are compared with the values of the control group by means of a t-test for independent samples. If the test assumptions are violated, non-parametric test methods such as the Wilcoxon rank sum test can be used for independent samples.
3. **Factors influencing the acceptance and experience of telemedical genetic counseling**
  In cooperation with the Hannover Center for Health Communication [HC]^2^ of the HMTM Hannover (Hanover University of Music, Drama and Media), further factors that influence the use and experience of (telemedical) genetic counseling are evaluated. The factors and associated dimensions are: evaluation of telemedical or personal genetic counseling, motivation of the use, trust in genetic counseling, performance and effort expectancy, impact of individualization and synchronicity, influence of habits, eHealth literacy, social norms, facilitating conditions and risk perception. For this purpose, adaptations of validated questionnaires are used. The values of the intervention group are compared with the values of the control group by means of a t-test for independent samples. If the test assumptions are violated, non-parametric test methods such as the Wilcoxon rank sum test can be used for independent samples.
4. **Satisfaction of oncologists and medical geneticists with the structured pathway**
  The satisfaction of oncologists with the structured pathway is qualitatively examined within a focus group with six to eight participating oncologists (participation in this is agreed in the cooperation agreement with the oncologists). The qualitatively collected data are transcribed verbatim and evaluated according to predetermined criteria in terms of content analysis using the qualitative data analysis program MAXQDA. The analysis procedure is based on the basics of structuring qualitative content analysis according to Mayring [13]. The results of the focus groups are used to create a questionnaire for the quantitative evaluation and will be used in the other clusters. Three topics are examined individually: (1) The support of the decision-making about the suspected TRS-diagnosis; (2) The preparation for the personal consultation; and (3) The following process as well as the strengthening of the capacity to relate to the patient and its family members. In addition, the satisfaction of oncologists with the didactic and content-related preparation of the training courses is evaluated by questionnaires.
  In order to determine satisfaction of medical geneticists with the processes, telephone-based qualitative individual interviews are carried out. In addition, both in the control and in the intervention phase, the burden of the respective consultation is collected by means of the NASA-TLX questionnaire. The National Aeronautics and Space Administration Task Load Index (NASA-TLX) [14] is a multidimensional survey for the subjective measurement of workload. There is a German adaptation for recording the workload of medical consultations.
5. **Cost-effectiveness of genetic counseling**
  The cost-effectiveness of genetic counseling is operationalized as a ratio of incremental intervention costs compared to routine care and the additionally identified TRS patients. When calculating the costs per genetic consultation, different perspectives are considered. *Patient perspective:* Costs on the part of the patients include direct costs for the use of the consultation (journey or transport to the genetic consultation) as well as indirect costs in the form of productivity loss due to the time spent (e.g. round trip, consultation). These costs are recorded by means of questionnaires. *Service provider perspective:* In addition, the costs of service provision are estimated. This includes, in particular, the costs of the working time spent on the preparation, implementation and follow-up of the consultation with the respective service providers. The cost per genetic counseling is compared between the intervention group and the control group for each perspective.

### Legal basis for the new form of care

As a legal basis for the new form of care, the parties involved in the project (consortium management and participating oncology practices) conclude cooperation agreements in accordance with § 92a of the German Social Code, Book Five (SGB V), in which the project components and contents are agreed, responsibilities defined and billing determined. The statutory health insurance AOK Niedersachsen supports the preparation of these cooperation agreements and ensures compliance with SGB V conformity. The additional work of the participating oncologists is compensated by an extra reimbursement. The enrollment of the insured persons takes place in the practices participating in the project. Prior to enrollment, each patient must be informed by the oncologists about the nature, significance and objectives of the OnkoRiskNET project as well as the type and scope of the documentation. The project complies with the applicable legal provisions including data protection requirements. The genetic testing is carried out as part of the standard care and is only carried out after appropriate information in accordance with § 9 of the German Genetic Diagnostics Act and consent.

The patient may revoke his or her consent at any time with effect for the future in writing or orally to the responsible medical person. If the revocation is made orally, it must be documented immediately. The responsible medical person guarantees the patient and affected family members at any time (in accordance with § 7 “proof of revocation” as well as § 11 “communication of the results of genetic tests and analyses”) the right to non-knowledge of the patients by withdrawing the consent to the genetic analysis. The patients can decide that examination results should be destroyed at any time.

Personal information about the participants will be collected in the Departments of Human Genetics Hannover and Dresden according to the General Data Protection Regulation in Germany (DSGVO). Furthermore, contracts about collection, sharing and storage of patient data during and after the trial were concluded between the HMTM Hannover or the institute Inav or the Department of Human Genetics of the University Hospital Carl Gustav Carus Dresden and the Department of Human Genetics of Hannover Medical School.

### Transferability of the results

The project is suitable as a model for at least nationwide, in principle European wide care of oncological patients with a suspected genetic tumor risk syndrome. The necessary structures are available at nearly every Department of Human Genetics of university hospitals, but also in large medical genetic practices and laboratories in Germany. Our approach aims to improve care in structurally weak regions, but can also be applied to care in metropolitan areas.

In oncological care, there is a clear trend towards the increased use of molecular genetic diagnostics for the use of targeted therapies. In this context, either primary genetic TRS are identified in affected patients or a TRS is identified on the basis of tumor sequencing. The new form of care would also be ideally suited for the further care and consultation of these patients. Furthermore, the number of diseases for which a genetic cause can be identified is increasing. Our model can serve for collaborations between all groups of specialised physicians and medical geneticists for the care of patients affected by a genetic disease. In order to prove the transferability of the findings in this project, the above-mentioned intervention will be extended to the health care region of the consortium partner University Hospital Carl Gustav Carus Dresden in Saxony.

## Discussion

The new form of care aims to improve the care of patients with a tumor risk syndrome and to facilitate access to genetic diagnostics. The study is intended to show possibilities of how comprehensive and interdisciplinary genetic patient care can be designed in the future using digitalization and networking in the health care system. In case of a positive evaluation and a positive statement of the Federal Joint Committee (G-BA), our aim is to implement the elements of our study for care of patients with tumor risk syndromes in the standard healthcare in Germany and extend it to all regions in Germany.

## Supplementary Information

**Additional file 1.**

## Data Availability

The data that support the findings of this study are available from the institute Inav (Institut für Versorgungsforschung, Germany) but restrictions apply to the availability of these data, which were used under license for the current study, and so are not publicly available. Data are however available from the corresponding author upon reasonable request and with permission of G-BA (Gemeinsamer Bundesausschuss, Germany).

## Abbreviations

GCSS: Genetic Counseling Satisfaction Scale
MICRA: Multidimensional Impact of Cancer Risk Assessment
NASA-TLX: National Aeronautics and Space Administration Task Load Index
PARP: Poly (ADP-ribose) polymerase
TRS: Tumor risk syndrome
VSQ: Visit-Specific Satisfaction Questionnaire
