# Supplementary material for: OnkoRiskNET: a multicenter, interdisciplinary, telemedicine-based model to improve care for patients with a genetic tumor risk syndrome
Source: BMC Health Serv Res. 2022 Jun 21;22:805. doi: 10.1186/s12913-022-08172-2 (PMC9210737; doi:10.1186/s12913-022-08172-2)
Supplement: Supplementary file 1 — Additional file 1. [file 12913_2022_8172_MOESM1_ESM.pdf]

Bitte für Etikett freihalten

EINWILLIGUNGSERKLÄRUNG

zur Teilnahme an der Studie "OnkoRiskNET"
Kooperationsnetzwerk zur wohnortnahen Versorgung von Patienten und Familien mit einem genetischen Tumorrisikosyndrom

Bitte lesen Sie den folgenden Text aufmerksam durch, kreuzen Sie Zutreffendes an und unterschreiben Sie die Einwilligungserklärung, sofern Sie damit einverstanden sind.

Ich bin durch den unten genannten Arzt über die Ziele, den Ablauf, die Bedeutung, den Nutzen und die Risiken, sowie die datenschutzrechtlichen Aspekte meiner Teilnahme an der o. g. Studie des Instituts für Humangenetik der Medizinischen Hochschule Hannover und des Instituts für Klinische Genetik des Universitätsklinikums Carl Gustav Carus an der TU Dresden aufgeklärt worden. Ich habe die diesbezügliche schriftliche Patienteninformation erhalten, diese gelesen und verstanden. Alle meine Fragen sind zu meiner Zufriedenheit beantwortet worden. Ich hatte genügend Zeit, um meine Entscheidung zu überdenken und frei zu treffen. Ich bin darüber aufgeklärt worden, dass meine Teilnahme freiwillig ist und ich meine Einwilligung ganz oder teilweise jederzeit ohne Angabe von Gründen widerrufen kann, ohne dass mir daraus irgendwelche Nachteile entstehen, dass jedoch die Rechtmäßigkeit der bis zum Widerruf erfolgten Nutzung der Daten davon jedoch unberührt bleibt. Mir ist bewusst, dass im Falle des Widerrufs die Daten gelöscht werden.

Ich willige ein, dass meine im Rahmen der Studie OnkoRiskNET erhobenen Daten, wie in der Patienteninformation beschrieben, erhoben und gespeichert werden und für die genannten medizinischen Forschungszwecke ausgewertet werden.	☐ Ja	☐ Nein
Ich willige dem Austausch meiner Daten zwischen allen Kooperationspartnern der Studie, wie in der Patienteninformation beschrieben, ein.	☐ Ja	☐ Nein
Ich willige ein, dass das Institut für angewandte Versorgungsforschung Berlin mich für telefonische Interviews, wie in der Patienteninformation beschrieben, kontaktieren darf.	☐ Ja	☐ Nein
Ich willige ein, dass eine Verknüpfung meiner Daten mit denen meiner Familienangehörigen erfolgen darf, damit die Familienzugehörigkeit für die Auswertung der Studie festgestellt werden kann.	☐ Ja	☐ Nein
Ich willige ein, dass eine Verknüpfung meiner Person mit dem /der mich behandelnden Onkologen / Onkologin für die Auswertung der Studie erfolgen darf.	☐ Ja	☐ Nein
Ich willige ein, dass ich evtl. zu einem späteren Zeitpunkt erneut kontaktiert werde		
• zum Zweck der Gewinnung weiterer Daten.	☐ Ja	☐ Nein
• zum Zweck einer Erweiterung meiner Einwilligung.	☐ Ja	☐ Nein
• Zum Zweck einer Nachverfolgung meines Krankheitsverlaufes und Einflussfaktoren auf meinen Gesundheitszustand, wie in der Patienteninformation beschrieben.<br><br>Diese Rückmeldungen sollen erfolgen über das Institut für Humangenetik bzw. für klinische Genetik, in welchem meine Daten gewonnen wurden. Alternativ wünsche ich, dass folgender Arzt die Informationen erhält und mich informiert (bitte angeben):	☐ Ja	☐ Nein

Name und Anschrift des/der Arztes/Ärztin:<br><br>…………………………………………………………………………………………	

Datenschutzrechtliche Einwilligung:
Ich bin über den Zweck, den Umfang und die Dauer der personenbezogenen Datenverarbeitung sowie die möglichen Empfänger meiner Daten mündlich und schriftlich informiert worden. Mir wurden die für Datenverarbeitung verantwortlichen Personen und deren Kontaktdaten genannt. Mir wurden die Kontaktdaten der zuständigen Datenschutzbeauftragten genannt. Ich wurde über meine Rechte hinsichtlich des Datenschutzes informiert.
Ich willige ein, dass Mitarbeiter der mich betreuenden klinischen Einrichtung Einblick in meine Original-Krankenunterlagen nehmen und Befundkopien archiviert werden. Ich willige weiterhin ein, dass meine medizinischen Daten zentral an das Institut für Humangenetik der Medizinischen Hochschule Hannover weitergegeben werden und dort gespeichert werden. Ich stimme zu, dass meine medizinischen und studienrelevanten Daten in pseudonymisierter Form auf den Servern der Medizinischen Hochschule Hannover, des Instituts für Klinische Genetik am Universitätsklinikum Carl Gustav Carus der TU Dresden, dem Institut für Journalistik und Kommunikationsforschung der Hochschule für Musik, Theater und Medien Hannover (HMTMH), der Tivian GmbH Köln und/ oder dem Institut für angewandte Versorgungsforschung gespeichert werden.
☐ Ja ☐ Nein

Des Weiteren ermächtige ich hiermit die für mich zuständigen Onkologen / Onkologinnen, mit dem Institut für Humangenetik der Medizinischen Hochschule Hannover und/oder dem Institut für Klinische Genetik des Universitätsklinikums Carl Gustav Carus der TU Dresden Daten zu allen bekannten und meine Person betreffenden Tumorerkrankungen mit detaillierter Diagnose, Tumorstadium, Lokalisation, Therapiemethoden, -verlauf und- abschluss, sowie den weiteren, in der Patienteninformation genannten personenbezogenen Daten auszutauschen.
☐ Ja ☐ Nein

Sofern ich Befunde von Angehörigen mitgeschickt/ vorgelegt habe oder Angaben über Angehörige gemacht habe, erkläre ich, dass ich von meinen Angehörigen befugt bin, diese dem Institut für Humangenetik bzw. Institut für Klinische Genetik zur Verfügung zu stellen:
☐ Ja ☐ Nein

Eine Kopie der Patienteninformation (inkl. Informationen zum Datenschutz) sowie der Einwilligungserklärung habe ich erhalten. Das Original der Einwilligungserklärung verbleibt bei der mich behandelnden klinischen Einrichtung.

Bitte in Blockschrift ausfüllen:

Name, Vorname:	Geburtsdatum:
Straße/Hausnummer	Postleitzahl/Wohnort
Telefon- und/oder Handynummer	E-Mail-Adresse
Ort/Datum	Unterschrift des/der Ratsuchenden
Datum/ Aufklärende(r) Arzt/Ärztin (Stempel)	Unterschrift aufklärende(r) Arzt/Ärztin
